# Legislatively Excluded, Medically Uninsured and Structurally Violated: The Social Organization of HIV Healthcare for African, Caribbean and Black Immigrants with Precarious Immigration Status in Toronto, Canada

**DOI:** 10.1177/10497323221082958

**Published:** 2022-04-05

**Authors:** Apondi J. Odhiambo, Lisa Forman, LaRon E. Nelson, Patricia O'Campo, Daniel Grace

**Affiliations:** 1 7938 University of Toronto, Toronto, ON, Canada; 2 5755 Yale School of Nursing, New Haven, CT, USA

**Keywords:** Canada, healthcare, HIV, immigrants, legislation, precarious immigration status, medically uninsured, qualitative research, institutional ethnography, structural violence, health equity, Social justice

## Abstract

African, Caribbean and Black immigrants face persistent legislative barriers to accessing healthcare services in Canada. This Institutional Ethnography examines how structural violence and exclusionary legislative frameworks restrict the right to HIV healthcare access for many Black immigrants. We conducted semi-structured interviews with Black immigrants living with HIV (*n* = 20) and healthcare workers in Toronto, Canada (*n* = 15), and analyzed relevant policy texts. Findings revealed that exclusionary immigration and healthcare legislation shaping and regulating immigrants’ right to health restricted access to public resources, including health insurance and HIV healthcare and related services, subjecting Black immigrants with precarious status to structural violence. Healthcare providers and administrative staff worked as healthcare gatekeepers. These barriers undermine public health efforts of advancing health equity and ending HIV “while leaving no one behind.” We urge continued policy reforms in Canada’s immigration and healthcare systems regarding HIV care access for Canada’s precarious status immigrants.

## Introduction

Canada has one of the highest rates of immigration in the world, with a steady stream of Black immigrants from sub-Saharan African and the Caribbean countries (Statistics Canada, 2019). HIV disproportionately impacts African, Caribbean, and Black (ACB) communities in Canada (Mbuagbaw et al., 2020b). Although Black immigrants represent only 3.8% of the general Canadian population, they account for approximately 14.9% of people living with HIV (Haddad et al., 2019; PHAC, 2020). In this article, we will use the terms ACB immigrants and Black immigrants interchangeably. We recognize the diversity and heterogeneity of this population in terms of ethnicity, nationality, race, sexuality, and immigration status.

Provision and access to HIV healthcare and anti-retroviral treatment (ART) are core determinants of health for people living with HIV (Assefa et al., 2020). Evidence shows that timely, high quality and consistent access to healthcare and treatment by people living with HIV is crucial for reducing viral load to undetectable levels, maintaining and improving health outcomes and wellbeing, and preventing onward transmission of HIV (Assefa et al., 2020; Cohen, 2011; Cohen et al., 2020). This scientific evidence informed the development of the UNAIDS 90-90-90 targets, which stated that by 2020, 90% of all people living with HIV will know their HIV status, 90% of people with an HIV diagnosis will receive ART, and 90% of people receiving ART will achieve viral suppression, and the Fast Track strategy which aimed to reduce incidences of HIV infection and end the epidemic by 2030 (Cohen, 2011; UNAIDS, 2014a). Despite biomedical advances and global initiatives aimed towards ‘ending HIV’, ACB immigrants in Ontario are being left behind in the HIV response due to inequities that restrict their right to health, including access to HIV healthcare and health-related services, leading to poor health outcomes (Etowa et al., 2020; Mbuagbaw et al., 2020b).

## Laws, Inequities, Structural Determinants of Health, and Right to Health

Laws are determinants of health, primarily through the right to health which includes access to HIV health-related services (Gostin, 2021). The right to the highest attainable standard of health is recognized in several human rights treaties internationally, many of which Canada has ratified and is legally bound by. Article 25(1) of the Universal Declaration of Human Rights (UDHR) states that “Everyone has the right to a standard of living adequate for the health and wellbeing of himself and of his family, including…medical care and necessary social services, and the right to security in the event of…sickness” (UNGA, 1948). Under Article 12 of the International Covenant on Economic, Social and Cultural Rights (ICESCR), it is generally expected that Member States of the UN meet their commitment to support and ensure the right to health and basic healthcare access, especially in realizing their treaty obligations. Although Canada is a country that prides itself on multiculturalism and a humanitarian approach to immigration and views its “universal” and “equitable” access to healthcare as an intrinsic value, its legislation does not grant the right to health for people with precarious immigration status.

Immigrants with precarious immigration status refer to individuals who are institutionally labeled as foreign nationals with less-than-permanent legal status and who might experience shifts between legal and illegal status in Canada (Goldring et al., 2009). Under subsection 20.1(2) of the Immigration and Refugee Protection Act (IRPA), a foreign national is “a person who is not a Canadian, permanent resident and includes a stateless person” ("Immigration and Refugee Protection Act, 2001"). Goldring and colleagues use the term “precarious status” to highlight one’s legal and social positioning and institutionalized forms of precariousness in a socio-political setting (Goldring et al., 2009). It also accommodates those immigrants whose status is uncertain as they move in and out of different degrees of legal status, including those on 3 months wait imposed by the province of Ontario on people moving from out of province or those with newly acquired permanent residence status.

Canada’s immigration and health laws intertwine to restrict the right to health, including access to healthcare and health-related services for immigrants with “precarious immigration status” (Assari & Caldwell, 2018; Goldring et al., 2009; Hartog, 2020). The primary piece of legislation governing Canada’s healthcare insurance system at the federal level is the Canada Health Act (CHA) ("Canada Health Act, 1985”). Under CHA, the right to health and access to healthcare comes down to the question of legal immigration and residence status. The CHA defines those who are eligible for public health insurance as “a resident” of a province who is “lawfully entitled to be or to remain in Canada who makes his home and is ordinarily present in the province but does not include a tourist, a transient or a visitor to the province” ("Canada Health Act, 1985"). Residents of Canada who are entitled to access healthcare and health-related services are those meeting the conditions set in federal and provincial legislative frameworks as ordered by CHA. The condition of residency raises questions about inclusion, entitlement, and equity in health and access to healthcare. In this work, we use the term “medically uninsured” to refer to individuals excluded from accessing public healthcare coverage in Canada (Caulford & D'Andrade, 2012). The absence of the right to health, including healthcare for precarious status immigrants, is a growing concern (Campbell et al., 2014; Vyas et al., 2020). In a recent report regarding Canada’s implementation of the right to health, the UN Committee on Economic, Social and Cultural Rights (CESCR) expressed concerns about Canada’s denial of healthcare access for precarious status immigrants. (ICESCR, 2016).

Scholarship has highlighted the significance of legal immigration status as a structural determinant of health and the consequences of exclusionary and anti-immigration laws on immigrants’ health (Philbin et al., 2018). A growing body of knowledge on social determinants of health shows that immigration and health laws, policies and institutional practices, as well as extended state-level legislation and regulations, intersect to affect immigrants’ healthcare and treatment access, including access to HIV and treatment (Caulford & Vali, 2006; Li, 2017; Maria Elenade Trinidad Young MPH, 2018; Martinez et al., 2015; Motomura, 2014). There is an acknowledgment that Black immigrants experience barriers navigating the complex Canadian healthcare system to access HIV healthcare and treatment, improve their quality of life, and achieve optimal health (Logie et al., 2017; Mbuagbaw et al., 2020a). While precarious status immigrants face the most barriers, the socio-political context also influences the racialization of Black people who are documented or Canadian born and may experience anti-black racism and discrimination despite their citizenship or legal status while seeking care (Davda, 2019; García, 2017; Reitmanova & Gustafson, 2012). Immigration and health laws contain policies that determine rights, including the right to health, and deny certain populations access to healthcare based on the legal status, leading to harm and suffering, constitute structural violence.

### Structural Violence

Structural violence “emerges from the unequal distribution of power and resources” (Weigert, 2010, p. 126). Structural violence conceptualizes and describes the ways social relations such as laws, systems, policies and, institutional practices deny and block individuals, groups, and populations from realizing their full potential resulting in harm (Galtung, 1969). Structural violence which operates at multiple and intersecting levels are embedded into structures of social institutions, materializing as unequal power, inequitable distribution of resources, and eventually in inequitable and unjust life opportunities (Giddens, 1984). Price asserts that “[in] order to see violence, one must see the structures” (Price, 2012, p. 6). Similarly, Dilts and colleagues argue that rather than focusing primarily on “agents and intentions,” priority should be on violence “built into structures, institutions, ideologies, and histories” (Dilts et al., 2012, p. 191). By focusing on structure and its harmful effects, the concept of structural violence connects experiences across systems and contexts through which marginalized individuals and groups are subjected to patterns of violence. Violence in this context symbolizes the avoidable impairment of basic and necessary human needs by laws, policies, standards, and norms that penetrate and shape every aspect of our lives. Structural violence, therefore, reveals legal and institutional processes through which immigration and healthcare laws produce and normalize harm and suffering by seeming neutral but exclusionary and discriminatory practices. Galtung argues that violence is produced and perpetuated when a person dies “despite all the medical resources in the world,” (Galtung, 1969, p. 168). In this study, inequities and injustices experienced by immigrants based on one’s immigration, residence, and socio-economic status are conceptualized as violent structural effects of exclusionary immigration and healthcare systems, laws and intersecting institutional practices (Farmer, 2004; Galtung, 1969; Weigert, 2010). Several scholars have used structural violence to understand how socio-political structures and forces produce and perpetuate inequities that shape the “landscape of risk” and disproportionate impact marginalized communities (Hamed et al., 2020; Kelly, 2005, p. 721; Lane et al., 2004). Hamed et al. (2020) used the concept of structural violence to theoretically frame racism and explore how racism built in healthcare institutions and processes produce and sustain health inequities (Hamed et al., 2020).

There is a significant knowledge gap on how intersecting legislative frameworks and institutional practices shape access to HIV healthcare and treatment for Black immigrants with precarious immigration status, and the resultant impact on individual health outcomes and public health remains underexplored. This institutional ethnography (IE) uses the concept of structural violence to illuminate the “sometimes hidden and [increasing] violent [and harmful] effects” of laws, policies and institutional practices on Black immigrants with precarious status and living with HIV (Menjívar & Abrego, 2012, p. 1382). More specifically, this study examines how legislation regulating healthcare delivery and related institutional practices shape and constrain access to HIV healthcare and treatment for Black immigrants with precarious status in Toronto, Canada. It also explicates how legislative frameworks impact individual human rights, public health, and efforts to end HIV.

## Method

IE is a method of inquiry for investigating institutions from the standpoint of disempowered and marginalized groups (Smith, 1987). This alternative sociological tradition is concerned with mapping ruling relations and the role of institutional powers in producing, organizing, governing, and coordinating people’s local social worlds, knowledge, practices and activities (Ion et al., 2020b; Mykhalovskiy & McCoy, 2002). In IE, ruling relations are broad institutional, managerial, and professional pathways of governing and organizing society and our social world (Smith, 1987). The principal commitment of an IE inquiry is about “discovering ‘how things are actually put together,’ ‘how things work’” within an institutional complex by ruling relations (Ion et al., 2020a; Smith, 2006, p. 1). Rankin defines institutional complex as “the ruling practices that order contemporary Western societies” (Rankin, 2017, p. 2). In IE, an institution does not refer to a single system or agency but rather to collections of intersecting relations organized around a specific function, such as healthcare, legislation, or immigration (Hughes, 2014; Sinding, 2010). For example, McCoy used IE to investigate the organization of physician-based outpatient healthcare from the standpoint of women and men living with HIV and experiencing marginality (McCoy, 2005). We conceptualized the immigration system as an institutional complex producing precarious immigration status categories. Other scholars have used IE to examine the production of precarious status migrants and how the Canadian immigration system socially organizes precarious status queer and transgender migrants’ everyday lives (Lee, 2019). We also conceptualize the healthcare system as an institutional complex responsible for organizing and regulating healthcare delivery. In this study, we are concerned with how the institutional complexes of immigration and healthcare intersect to organize and shape access to HIV healthcare and treatment for precarious status immigrants.

Smith’s sociology for people begins with exploring the everyday life experiences, activities, and perspectives of a group of people or individuals concerning their engagement with an institutional complex and connecting them back to social relations that shape their activities within that institutional complex (Grace, 2019). In this study, we are concerned with how Black immigrants with precarious immigration status go about accessing HIV healthcare and medication in a social context where they have no constitutional right to healthcare.

IE includes analysis and understanding of Smith’s concept of “work,” which refers to anything that people do that “has an intent and requires time, effort, and skill and coordinated across time and space….[is] done under certain condition, and which included much more than only paid work” (Smith, 2005, pp. 151–152; Smith, 2006). Mykhalovskiy coined the concept of “healthwork” to investigate how people living with HIV navigate the healthcare systems to access ART in Canada (Mykhalovskiy, 2008; Mykhalovskiy & McCoy, 2002). In this study, “healthwork” is the experiential pathway to understanding what Black immigrants living with HIV do to access HIV healthcare and treatment within Canada’s healthcare system. “Healthwork” is analytically concened with the work extra local actors such as healthcare providers, administrators, and policymakers do in the delivery of HIV healthcare and treatment (Grace, 2019). In IE, texts also play an essential element. Texts are primarily legislative frameworks, organizational policies, and professional knowledge, guidelines, and policies (Smith, 1987). Texts coordinate institutional processes translocally and help to reveal how different relations govern daily activities and work in institutional settings such as healthcare. We examine legislative frameworks and institutional practices shaping and regulating healthcare delivery in Canada, including federal and provincial immigration and healthcare legislation, policies, and human rights frameworks.

### Study setting

Ontario is a valuable study setting for understanding how legislative frameworks and institutional practices constrain HIV healthcare and treatment, resulting in long-term harm and suffering for ACB communities and undermining public health and human rights. The 2016 Census shows that Ontario is home to approximately 52.4% of the total Black population in Canada (Statistics Canada, 2017). In 2016, Black people also represented 7.5% of Toronto’s total population, the highest proportion compared to other census metropolitan areas such as Montréal and Ottawa-Gatineau. In Ontario, Black immigrants account for 30% of HIV prevalence and 25% of new infections while representing only 4.7 of the general population (Mbuagbaw et al., 2020b).

### Sampling and Recruitment

The fieldwork was conducted between May 2019 and October 2020 following ethics approval from the University of Toronto Research Ethics Board. The first author recruited 20 key informants and 15 healthcare workers and policymakers for in-depth interviews. Purposive sampling techniques was used to identify and recruit Black people living with HIV (key informants), and healthcare workers and policymakers involved in the day-to-day formal and informal institutional processes of HIV care work (Campbell & Gregor, 2002M. Campbell & Gregor, 2002). The first author and the principal investigator in this study relied on community-based organizations (CBOs), AIDS service organizations (ASOs), shelters, HIV clinics, and clinical providers for recruitment support and referral of key informants. Key informants were included in the study if they self-identified as HIV positive, 18 years of age or older and Black immigrants from African and Caribbean countries. The recruitment of healthcare workers and health policy actors was iterative. Healthcare workers were requested to suggest other potential participants involved in the delivery of HIV healthcare. The first author contacted eligible participants until reaching saturation.

## Data Collection: Interviews with Black Immigrants and Health Workers and Policy Actors

This study relied on different data collection approaches, including interviews and textual analysis. We originally planned to conduct all interviews face-to-face. At the beginning of the COVID-19 pandemic and towards the end of data collection process, we received ethics approval to conduct the rest of the interviews by telephone or through zoom. All interviews were audio-recorded and transcribed based on participant consent.

### In-depth interviews

We first sought to understand Black immigrants’ (key informants) experiences of accessing HIV care. All key informants (*n* = 20) completed a demographic questionnaire to capture their social identities. We used semi-structured interview guides to conduct the interviews. The guide included questions about how key informants learned of their HIV status, how they accessed HIV healthcare and treatment, whom they interacted with as they sought care, what materials or documents healthcare workers provided, and what challenges they encountered accessing HIV healthcare and treatment. All interviews with key informants averaged 1.5 hours and were each compensated with $30 CAD honoraria.

Second, we examined the practices of the healthcare workers involved in the delivery of HIV care, including physicians (*n* = 4), HIV specialists (*n* = 4), social workers (*n* = 1), pharmacists (*n* = 1), frontline healthcare workers (*n* = 2), and health policy actors (*n* = 3) engaged in HIV-related work. Health workers experiential accounts helped us understand how their daily work of delivery healthcare are regulated and organized by laws, institutional practices and decisions made by administrators in extra-local settings (Balcom et al., 2021). We used a separate semi-structured interview guide to question healthcare providers: how they go about providing HIV healthcare to Black immigrants living with HIV; what texts regulate their work (e.g., legislative frameworks, guidelines, and protocols); what challenges constrain their work of providing HIV healthcare; and what workaround practices do they engage in to ensure Black immigrants access HIV healthcare. Policy health actors were interviewed using questions generated from policy concerns raised by Black immigrants and healthcare workers. Policy health actors were asked how different legislation and institutional practices identified by key informants and healthcare workers shape and constrain access to HIV healthcare for precarious status immigrants.

### Textual analysis

Dorothy Smith states that “texts are of central importance to institutional ethnography because they create this essential connection between the local of our (and others’) bodily being and the translocal organization of the ruling relations” (Smith, 2006, pp. 118–119). We analyzed pieces of legislation regulating healthcare delivery in Canada, such as the IRPA, CHA and Regulation 552 under the Ontario Health Insurance Act and related professional policies. We also reviewed policy documents organizing the provision of healthcare to medically uninsured people at the municipal level. Smith argues that “exploring how texts mediate, regulate and authorize peoples’ activities in modern societies expands the scope of ethnographic method beyond the limits of observation and interviews” (Smith, 2001, p. 159).

### Analytic Process and Mapping

Key informants’ transcripts were analyzed to understand the various forms of work that Black immigrants with precarious status undertook while negotiating access to HIV care. Informed by the legal framework of structural violence, the everyday practices of Black immigrants were mapped to identify and connect how institutional texts and practices organize and constrain their day-to-day work of HIV care. While reading key informant transcripts, we indexed practices and texts that were related to the work of accessing HIV care. Indexing involves identifying repetitive practices, discourses, or text while preserving the data’s materiality rather than classifying data into thematic categories (Rankin, 2017). Guided by issues identified by key informants as necessary in understanding their experiences accessing HIV care, we analyzed healthcare providers’ and health policy actors’ transcripts. We looked for areas of connections between Black immigrants’, healthcare workers’ and health policy actors’ experiences and identified texts and discursive materials organizing their work. After indexing was completed, the indexes were synthesized and reorganized into themes. The identified themes were categorized into institutional processes organizing HIV healthcare and treatment access for Black immigrants with precarious immigration status. Priority was given to legislative frameworks and institutional practices constraining and excluding Black immigrants from accessing HIV healthcare and workaround practices.

## Results

**Figure 1.**
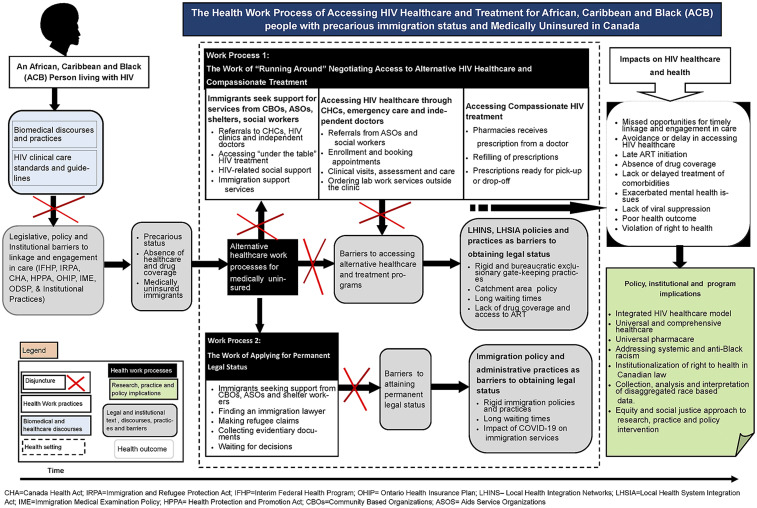
The health work process of accessing HIV healthcare and treatment for black people with precarious immigration status in Canada.

Our findings reveal the everyday work of accessing HIV healthcare for Black immigrants with precarious immigration status. We mapped how the institutional complexes of immigration and healthcare, and related legislation, policies and practices shape the everyday experiences of Black immigrants seeking HIV healthcare and treatment. More specifically, we uncovered the disjuncture between biomedical discourses and practices of HIV healthcare and treatment and Black immigrants’ experiences of precarity. We identified two work processes reflecting Black immigrants’ lived experiences of structural violence and its impact on HIV healthcare and treatment work. Figure 1 outlines complex work processes of how Black immigrants with precarious status who were legislatively categorized as “medically uninsured” and denied access to public healthcare services navigated their precarity to access HIV healthcare and treatment. The first work process highlights how Black immigrants negotiated and accessed HIV healthcare and treatment through patchy alternative programs (Figure 1, work process 1). The work process of applying for permanent legal status is foregrounded as the formal pathway to obtaining public healthcare coverage (Figure 1, work process 2).

## Precarious Immigration Status as Structural Violence and Barrier to HIV Healthcare

We began the inquiry by asking immigrants how they came to learn of their HIV positive status and how they went about accessing care. Some immigrants came to Canada with knowledge of their HIV-positive status. Others explained that they learned they were HIV positive through emergency department visits, hospitalization, or immigration medical examinations (IME) (Sanders & Bisaillon, 2019). At the core of key informants’ concerns was how to access HIV healthcare and medication as immigrants in Toronto. Black immigrants and healthcare workers identified precarious immigration status as a barrier to accessing HIV healthcare and medication in Toronto (Goldring et al., 2009). As a result of precarious immigration status, immigrants lacked access to public health insurance coverage and HIV healthcare. Black immigrants reported that they were expected to pay for HIV healthcare and medication through private insurance, out-of-pocket, or employment-based insurance. However, immigrants noted that they did not have adequate income and were precariously employed. One participant expressed that:

I did not have status [immigration] and medication in this country is extremely expensive…they [pharmacy] were quoting about [CAD] 1600, and that is before you have seen a doctor and got a prescription… So, it was going to cost me a lot… I did not have an income; I was just still in the shelter, and just getting even emergency medication was quite difficult in the beginning.

Healthcare providers also noted that medically uninsured individuals who decide to access healthcare through emergency visits or hospitalization are billed. However, Black immigrants lack money to pay the medical bills. A physician working in an HIV clinic explained that

When individuals with non-status go to the hospital and get registered…the hospital sends them a bill…And that is for just being registered as a hospital visit…That has nothing to do with the doctor’s visit. [However], these patients do not have the money to pay the bill.

The costs of paying out-of-pocket or buying private insurance to access HIV healthcare and prescription medication were prohibitive and significant barriers for all the immigrants we interviewed.

A textual analysis of legislation regulating healthcare access in Ontario revealed that access to publicly funded healthcare is through the Ontario Health Insurance Plan (OHIP) as provided by the General Regulation made under the Health Insurance Act (RRO 1990; Reg. 552) (CIC, 2010). Under section 1.1(1) of Reg. 552, an individual is deemed eligible to access coverage by being a “resident” of Ontario. However, under section 1.4(4) of Reg. 552, precarious status immigrants, including temporary visa holders, transient and visitors such as those in Canada on tourist visas after sponsorship relationships have ended, and undocumented residents are not eligible for these services. Immigrants who obtain permanent residence status are eligible to receive OHIP health benefits if they can prove they were residents in a province or territory for the duration of time supported in a jurisdiction’s legislation. Under Regulation 552, precarious immigrants also include immigrants with permanent resident status in the “three-month waiting period” for OHIP coverage. The 3-month wait period is a legislative text that applies to new legal immigrants to Ontario and returning permanent residents and Canadians who have been living outside of Ontario for more than 212 days in the calendar year (Caulford & D'Andrade, 2012; Goel et al., 2013). Immigrants who moved to Toronto as landed immigrants or from other provinces spoke of how their treatments were interrupted because they were required to wait for 3 months before they could be eligible to access public healthcare in Ontario. The immigrants were unaware of the “three-month wait” period policy before moving to Toronto. They learned of the policy either while trying to access HIV care or applying for a health card.

Whereas the delivery and provision of healthcare in Canada is primarily a provincial mandate, the federal government retains the constitutional responsibility to provide healthcare services to refugees on making a successful refugee claim. The federal government’s role in providing healthcare services to refugees is limited to the Interim Federal Health Program (IFHP) (CIC, 2018). The IFHP, which was established in 1957, is a unique federal fund under the IRPA that provides temporary and limited healthcare coverage to refugees and refugee claimants until they become eligible for healthcare coverage under provincial or territorial health insurance policy or leave Canada. Under these provincial and federal legislative conditions, immigrants without OHIP and IFHP are considered medically uninsured and therefore not eligible for public healthcare coverage (Caulford & D'Andrade, 2012).

### The Work of Gatekeeping Healthcare Access

Key informants and healthcare workers commonly reported that medically uninsured Black immigrants encountered institutional and administrative barriers when trying to access HIV healthcare within clinical and healthcare settings. Healthcare providers and administrative staff policed clinical and healthcare settings and acted as gatekeepers by enforcing arbitrary administrative rules and procedures that determined who could and could not access healthcare. Participants commonly identified health insurance cards as an institutional tool and text used by gatekeepers to regulate healthcare access. Health policies and institutional practice mandate that a valid health insurance card is presented to the gatekeepers at clinics and hospitals as proof of eligibility to access any form of healthcare. A frontline worker expressed that:

Some people know they are HIV positive and are yet to get HIV treatment. How will they go to the hospital, yet they do not have insurance? They do not have health coverage…They cannot walk to a hospital [and] see a doctor…Nobody will see them…What is the first question they will be asked in the hospital? Every time you go to the doctor’s clinic, the first thing they ask is OHIP or IFHP card.

Because of legislative barriers, healthcare providers generally performed the work of gatekeeping access to HIV healthcare and making referrals rather than delivering care to immigrants with precarious status. Providers explained that they find themselves in conflicted situations as the legislation and institutional practices restrict care provision to people without health coverage. Instead, providers refer medically uninsured patients to community healthcare centers for care. A physician reported that:

Because there are a lot of people [that are] not covered under public healthcare, it becomes challenging for healthcare providers to piece together how they [uninsured] are going to access proper healthcare. In such a situation, I generally send people [uninsured] to CHCs [community healthcare centres] depending on where they live in the catchment areas because part of their funding is to help people that do not have healthcare coverage.

Another physician based in an HIV clinic in a hospital explained that most hospitals have “partnerships with most CHCs in Toronto to waive facility fees or hospitals pay a facility fee for people [without healthcare coverage] to see the doctor and have laboratory work done.”

Healthcare workers noted that COVID-19 pandemic and concomitant inequities in existing and emerging health and public policies disproportionately affected Black immigrants in terms of incidence infection and access to health services. In recognizing the significance of everyone having access to COVID-19 healthcare services, the Ontario government temporarily announced that it was “waiving the three-month waiting period for Ontario Health Insurance Plan (OHIP) coverage. Additionally, the province will cover the cost of COVID-19 services for uninsured people who do not meet the criteria for OHIP coverage” (Edmonds & Flahault, 2021; MOHLTC, 2020b). Healthcare workers expressed concerns that medically uninsured immigrants still experienced barriers despite the waivers due to gatekeeping practices. A frontline worker who was interviewed near the beginning of the pandemic expressed that “though the government directed healthcare to be accessible to everybody regardless of immigration status, immigrants are still being asked to pay when they go to emergency, provide OHIP and insurance.”

### The Work of “Running Around” Negotiating Access to HIV Healthcare and Medication

#### Negotiating access to HIV healthcare

With restricted access to public healthcare, Black immigrants living with HIV workaround legal barriers by turning to informal networks such as community organizations for support and resources. Black immigrants reported that they learned from their peers and social workers that they could access HIV healthcare services through alternative programs such as CHCs, dedicated HIV clinics, walk-in clinics, and independent doctors. Health providers explained the role that CHCs play in healthcare delivery in Ontario. A medical practitioner based at a CHC explained that “the CHCs is part of Ontario’s healthcare system charged with taking care of [medically] uninsured.” This practitioner elaborated that CHCs are funded by the Ministry of Health and Long-Term Care (MOHLTC) through Local Health Integration Networks (LHINs) to provide pre-defined primary healthcare to marginalized community members such as precariously insured, non-insured and undocumented people. Ontario’s LHINs are regional health authorities responsible for planning, integrating, and funding healthcare services within the province (MacLeod, 2015). The Local Health System Integration Act (LHSIA), 2006, SO. 2006, c. 4 requires that the LHIN and the CHCs enter into a service accountability agreement to enable LHIN to provide funding to CHCs for the provision of healthcare services ("Local Health System Integration Act, 2006"). A clinician working in a CHC explained

HIV-positive immigrants connect with CHCs because they need medication. Frontline workers based at CBOs and ASOs serving mostly immigrants and refugee populations refer Black immigrants to CHCs. Non-status immigrants sometimes access CHCs independently.

While the alternative programs were assumed to be accessible to medically uninsured residents in Toronto, Black immigrants revealed that they lacked knowledge of navigating the system to access the services. They explained that they started “running around” looking for ways to work around the complex and exclusionary policies and administrative processes to access alternative HIV healthcare programs. Black immigrants reported relying on social workers based in CBOs, ASOs, and shelters to help them navigate the system and access services. Immigrants expressed that the process of accessing social support through community organizations was long and emotionally draining. Participants described how they made numerous appointments, disclosed their HIV positive status to several social workers, and had to wait long before getting linked with independent doctors or medical practitioners working in CHCs. As a result of the emotional work of disclosing HIV positive status to several people, immigrants were subjected to trauma and fear. One immigrant expressed

For me, the biggest challenge was to have to disclose to so many people before getting support…I had to disclose to my shelter nurse, my shelter case manager…I had to go to agency X [blinded] and had to disclose to two people…Then I had to disclose to the person at agency Y [blinded] …Then the doctor. They finally sent me to a pharmacy that deals with people who have HIV…So that it seven people before getting service. And you know the disclosure process is not easy.

Black immigrants and healthcare providers commonly expressed that legislation governing CHCs and related institutional practices presented barriers to timely HIV healthcare. Immigrants described that CHCs have rigid eligibility criteria, tedious paperwork, long waiting lists, long waiting times during clinic days, and no secure and private waiting areas for HIV-positive people. As a result, immigrants reported experiences of fear, shame and stigma waiting in open areas. One immigrant expressed

Always when sitting [waiting], there is that fear because it is an open sitting area. There is always that fear of who is seeing me…What do they know about this clinic? What does it mean for them to see me here?

Healthcare workers noted a discrepancy in the institutionalization of CHCs as a workaround strategy to addressing healthcare gaps for the medically uninsured. They highlighted systemic barriers that constrain their capacity to provide HIV healthcare to their clients. The healthcare workers attributed the challenges faced by Black immigrants seeking HIV healthcare services within the CHC model of care to inadequate funding, limited healthcare providers, and the rule of catchment areas. The rule of “catchment areas,” which organize and govern CHCs’ operations, was reported as creating funding and institutional barriers to healthcare provision. Under section 14.1 (1) of the LHSIA, catchment areas refer to geographical areas of the health system networks set out on the LHINs map. A clinician expressed that “when CHCs are funded through regions or catchment areas, what this means is that together with their clients, they [CHCs] must fight for the same resources allocated to them.” Clinicians based at the CHC generally articulated that CHCs prioritize people based on catchment areas and place those who do not necessarily come from the catchment area on waiting lists. A clinician explained that “the CHCs conduct structured intake processes guided by the waiting lists.” The clinician further elaborated that the intake process requires individuals to “fill some forms” that include “demographic information about their identity-ethnicity, sexual orientation, gender, income, migration status, housing income, geographical location.”

Frontline workers making referrals for non-status persons or those doing intakes at CHCs explained that sometimes they have to plead an individual’s cases to the CHC’s manager because they cannot directly access care. It is only then that the CHCs can make someone a client and put them on a schedule. The requirement that agencies must plead the cases of people seeking care at CHCs creates another barrier to accessing HIV healthcare and treatment for HIV-positive immigrants already experiencing exclusion because of their precarious status.

Key informants and healthcare workers alike expressed that Black immigrants delay or avoid seeking HIV healthcare because of the complex and bureaucratic organization of the healthcare system, the lack of knowledge on navigating the patchwork system set-up for the medically uninsured, serial disclosures of HIV status and stigma. Furthermore, Black immigrants who accessed healthcare through emergency visits or walk-in clinics commonly expressed that they felt mistreated, stigmatized, and received inadequate treatment. Additionally, they expressed concerns about the long wait times at emergency care departments and the lack of comprehensive care. Immigrants who were hospitalized during emergency visits reported being discharged without an HIV healthcare plan and medication. Black immigrants felt that they experienced suboptimal clinical care and overall health management because of their Black race, HIV-positive status, and precarious immigration status. Immigrants who were in the process of regularizing their precarious immigration status by making refugee claims avoided accessing HIV healthcare because of fear of disclosure of their HIV-positive status, exposure to immigration authorities and deportation. A health worker based at an ASO explained:

There are people who have died, ended up losing their feet or eyes because of the amount of time it took for someone to talk to them…Maybe it is too late for them to get the care they deserve or need at the time.

#### Seeking access to HIV medication through compassionate drug programs

Healthcare providers and immigrants commonly identified lack of drug coverage as a shortfall of Canada’s public healthcare system and CHC’s care model and a critical missing component of HIV healthcare. Canada does not have a national pharmacare program; however, individual provinces and territories have independent public drug insurance programs (Demers et al., 2008; Yoong et al., 2015). In Ontario, people are expected to pay for medication out of pocket or through private insurance. However, individuals without private insurance can access drug coverage through multiple conditional programs (MOHLTC, 2020a). The Ontario Drug Benefit (ODB) program provides coverage to “residents.” However, access is limited to individuals who are eligible for OHIP and are either age 65 years and older, have low income, are receiving social assistance such as Ontario Works (OW) or Ontario Disability Social Program (ODSP), or are enrolled in the Trillium Drug Program (TDP) (MOHLTC, 2020a). The TDP is an annual provincial government program that provides drug benefits to Ontario residents with valid OHIP and who spend a large portion of their annual income on prescription medication. Healthcare providers noted that immigrants do not register for TDP because they are precariously employed, ineligible, or unable to pay the deductible, which is equally expensive. Furthermore, HIV medication is generally expensive and unaffordable for Black immigrants with precarious status.

Black immigrants and healthcare providers collectively reported that medication access is usually through compassionate drug programs provided by pharmaceutical companies and facilitated by ASOs or CHCs. One immigrant explained “If you don’t have a health card but get a good doctor, he can try to help you get people to write a letter for you to go to the pharmacy and give you medication.” Healthcare providers discussed that compassionate care involves providing medication to people who have no HIV treatment because they have no drug coverage and are unable to afford medication. A physician indicated that “people without drug coverage are able to access HIV medication through compassionate drug program on an interim while navigating other challenges…Maybe they are still looking for immigration papers or have lost their employment or are applying for trillium.” Temporary visa holders, such as international students who might have private insurance that does not cover everything, can access medication through local ASOs or compassionate programs that offer treatment for shorter periods.

Whereas some pharmacies provide compassionate medication to bridge the system’s gap, healthcare providers were concerned about “sustainability.” They questioned what would happen if the compassionate programs ended. One physician expressed that “there are some companies that do not want to give free medication forever” and therefore only make provisions “for six months to a year.” The local ASOs also do not have all HIV medications. Healthcare providers commonly identified the requirement that individuals must provide a “prescription” from a registered medical doctor to access HIV medication as a significant barrier facing compassionate care programs. Drug companies, pharmacies, and ASOs dispensing compassionate treatment require that individuals present a written prescription from a doctor before they can dispense compassionate medication. A frontline worker explained

What this means is that those who do not have a doctor cannot access compassionate medicine. Therefore, frontline healthcare workers have developed strong relationships with health providers and nurses who can write a prescription for non-status to access compassionate medication.

A key informant detailed how a social worker based at a local agency serving immigrants and refugees living with HIV managed to refer him to an HIV specialist even without healthcare coverage to get a prescription:

This person could refer me to get a specialist at the clinic who was able to start me right away on meds without me having any coverage at that point. He gave me a prescription and sent me to a specific pharmacy where I could get the meds. He took my blood.

Healthcare providers noted that immigrants who come to Canada knowing their HIV status and are already on ART encounter challenges accessing compassionate HIV medication because of lack of access to clinical care and laboratory assessments such as drug toxicity or resistance. These healthcare providers explained that people already taking ART must undergo drug toxicity and resistance assessment before they are started on different treatment regimes. Providers also noted that immigrants coming from countries such as South America and Africa come to Canada with HIV medications that are not available nor are recommended in Canada. For example, a healthcare provider explained that the medications that Black immigrants come with are sometimes “generic medication that is no longer recommended for use in Canada.” A frontline worker who also identified as a Black immigrant living with HIV explained

For medications not available in Canada, it means they [newcomers] must go through the resistant test, go through viral load and CD4 test and for the doctor to prescribe any new medication. it is not easy… It is complex, and it is how one negotiates with the healthcare provider. Sometimes it involves talking to CHCs and getting people seen urgently by a doctor, go through those tests to access treatment quicker.

A frontline worker explained that they engage in the work of “collecting medication from people that have medication and are not using and giving them under the table [informally] to those who need them.”

### The Work of Applying for Permanent Legal Status in Canada

Black immigrants and healthcare providers commonly reported that applying for permanent legal status in Canada was the only formal pathway for HIV-positive medically uninsured immigrants to obtain healthcare coverage and access sustainable HIV care. Black immigrants indicated that they lacked knowledge of how immigration and healthcare systems work and the potential impact of their immigration status on their day-to-day lives before coming to Canada. Therefore, most Black immigrants decided to apply for legal status only after arriving in Canada and encountering legislative barriers to accessing HIV healthcare. Immigrants imagined that having a legal status would make their lives living with HIV easily manageable. With legal immigration status, Black immigrants hoped that they would easily and quickly get an HIV doctor, access HIV care, and apply for the ODSP. An immigrant who came to Canada as a temporary worker and stayed after his work permit expired explained that he was diagnosed with HIV during emergency hospitalization. He elaborated that he was not granted HIV healthcare during his hospitalization and was discharged from emergency care without a healthcare plan, referral, or HIV medication because of his precarious status. This immigrant noted that because he had no financial capacity to pay for healthcare and treatment out-of-pocket, he had no choice but to claim refugee, hoping that gaining legal immigration status would grant him access to healthcare coverage.

Healthcare providers working in public hospital settings explained that many immigrants do not have healthcare coverage for many reasons. A physician explained that: “Immigrants come to Canada on a visitor’s visa or student’s visa, and when their visa expires, they decide they want to live in Canada.” Healthcare providers expressed that it is daunting to provide HIV care to precarious status immigrants without healthcare coverage. A physician explained that “it becomes exceedingly difficult for both the immigrants and healthcare providers in such cases because it becomes an immigration issue that must be addressed through immigration. Therefore, all roads lead to immigration if you ask me.” A healthcare provider expressed that “A big factor in HIV healthcare and treatment is making sure people who do not have any healthcare coverage get secure and ongoing medication.” Several healthcare providers emphasized that the immigration work of applying for legal permanent status or “making refugee claims” is a priority for precarious status immigrants living with HIV.

At the time of the interviews, some immigrants had not started the refugee application process and were preparing to apply. Others were working on their claims, while others were awaiting decisions on their request. These participants commonly identified a lack of knowledge and resources as barriers to applying for legal status or making refugee claims. Immigrants relied on social workers based in shelters, CBOs and ASOs to get immigration lawyers. One key informant elaborated

I went to the shelter, and they started asking me questions about how I came here. Shelter people helped me to get a doctor and a lawyer. I started to get some papers [refugee application] …They got a translator for me, they asked me questions, and I told them my story. The lawyer told me; my workers will take me to the immigration office. I started preparing myself, and the lawyer asked me questions, and I told my whole story to the lawyer. They gave me a date to go to court, and so I went to court.

Black immigrants and health policy actors reported that bureaucratic and complicated immigration systems and changes in immigration processes constrained and delayed their obtaining permanent legal status and, ultimately, healthcare coverage. Participants expressed that the institutional process of applying for and proving the authenticity of refugee claims was complex and challenging. They noted that the immigration legislation regulating refugee application processes mandated applicants to provide extensive evidence to support claims. An immigration lawyer based at a local CBO explained that the process required: “people to complete a lot of paperwork, have a lawyer organize and sign the paperwork in addition to numerous immigration appointments.”

Black immigrants raised concerns about the waiting time, which added up to months and years of waiting for decisions about their claim applications and led to delays in getting healthcare coverage and accessing timely healthcare and social support, including the Ontario Works and ODSP. A physician expressed that

This period of waiting is the most challenging time for most people who come to Canada knowing their HIV status…Most people run out of medication, they do not have income, do not have a doctor, and if they fall ill within that time, too bad for them, they will end up in an emergency and still be charged [billed] or stay at home sick without access to care and medication.

Immigrants who had come to Canada with a few months’ supply of HIV medication explained that they feared their HIV medication would start running out before their refugee claims were accepted and became eligible for healthcare coverage. Precarious status immigrants expressed ambivalence about seeking HIV care because they felt healthcare providers might expose their HIV status, jeopardize their application for legal status and subject them to risk of being deported. These participants preferred to regularize their immigration status first before they could seek HIV care. One participant who had made a refugee claim stated: “I still have an immigration case going on. I do not know who will have access to the medical information.”

Black immigrants, healthcare providers, and health policy actors expressed how the novel Coronavirus (COVID-19) pandemic and the public health response has significantly affected all facets of government systems, including immigration work and the healthwork of looking for HIV healthcare and treatment. A frontline worker reported that

Undocumented people and refugee claimants who arrived in Canada before COVID-19 and other people trying to submit their application before lockdown are in limbo because they cannot see the [immigration] doctor to complete the paperwork…And without completing the paperwork, they cannot get Ontario Works, IFHP and apply for ODSP.

## Discussion

This IE inquiry examined how legislative frameworks and institutional practices shape and constrain access to HIV healthcare and treatment for Black immigrants through the lens of structural violence. The analysis started from the experiences of Black immigrants living with HIV and seeking HIV healthcare and treatment. Mapping the experiential accounts of Black immigrants problematized the discourses and practices of HIV healthcare and treatment. The lived experiences of Black immigrants in accessing HIV healthcare and treatment illustrate how structural violence is embedded in Canada’s social context and enacted through everyday interaction with laws, policies, and institutional practices. Scheper-Hughes argues that structural violence is “the ease with which humans are capable of reducing the socially vulnerable... into expendable non-persons” (Scheper-Hughes, 2004, p. 14). The findings indicate that structural violence embedded in Canadian laws, policies and institutional practices produce and perpetuate inequities, injustices, division, exclusion and discrimination that determine individual rights and entitlements including the right to health and who is entitled to human dignity through institutionalized and normalized everyday practices (Hamed et al., 2020). Farmer argues that violation of human rights is not coincidental or unintentional, but rather are symptoms of deeply rooted pathologies of power related to social relations, which influence who suffers and who is shielded from suffering (2003).The harmful effect of structural violence renders marginalized populations, in this case Black immigrants, dispensable and voiceless, leading to violation of human rights, “unequal life chances” and poor health outcomes (Uvin, 1998, p. 105). Thus, by mapping the individual experiences of Black immigrants entrenched in the “larger social matrix,” we established a contextualized understanding of how “large-scale social forces…translate[d] into personal distress and disease” (Farmer, 1996, pp. 261-262; Hamed et al., 2020).

The individual experiences of Black immigrants are embedded in the legal, social, and political context of Canadian “immigration exceptionalism” characterized by exclusionary, discriminatory, and punitive laws and policies. Black immigrants identified immigration law and institutional practices as essential determinants of right to health, including access to public health insurance coverage and healthcare services in Canada (Campbell et al., 2014). The study revealed that Canada enforces exclusionary immigration and health legislation, policies, and institutional practices that create a disenabling and discriminatory environment for Black immigrants seeking HIV healthcare and treatment. Exclusionary immigration laws defining immigrants’ categories and regulating the process of obtaining permanent legal status “other” Black immigrants to positions of precarious immigration statuses (Hughes, 2014). Othering, a process that “serves to mark and name those thought as different,” defines and privileges dominant identities by socially distancing and discriminating an (other) (Weis, 1995, p. 17). Exclusionary othering based on immigration status as a form of structural violence materializes through institutional practices of “marginalization, disempowerment and social exclusion,” leading to the borderline between “us” as in precarious status immigrants with no right to health, and “them” as in Canadian residents with the right to health (Bailey, 2008, p. 1). IE directed us to examine the material conditions and experiences of Black immigrants as “othered” and the injustices they faced trying to access HIV healthcare and treatment. We mapped out how legislative frameworks and institutional practices governing healthcare delivery in Canada “othered” Black immigrants with precarious status, creating conditions that excluded them from accessing HIV healthcare and treatment (Campbell & Gregor, 2002M. Campbell & Gregor, 2002). 

Findings show the fundamental role of immigration status as a social condition that determines the right to health and shape access to healthcare and health-related resources. We came to understand a two-tiered and complex patchwork healthcare system of exclusionary and unjust laws and institutional practices that exclude Black immigrants with precarious immigration status from accessing HIV healthcare and treatment within the public healthcare system: a public healthcare system that serves medically insured Canadians with the right to health, and an alternative system that caters for “medically uninsured” individuals with no right to health. Although Canada prides on a universal public health insurance system built on equity and solidarity, it enforces legislation and institutional practices that restrict the right to health, including access to medically necessary healthcare services for people with no legal permanent immigration and residence statuses ("Canada Health Act, 1985").

Black immigrants’ experiential accounts were packed with narratives of legal, institutional, and medical exclusion and violence, including experiences of discrimination, anti-Black racism, financial insecurity, lack of right to health, suboptimal access and fear of deportation and criminalization. We unmasked a multi-axial model of structural violence and suffering for Black immigrants with precarious immigration status and living with HIV. Farmer refers to “multi-axial model of suffering” as to how individual’s day-to-day realities intertwin with broader structures and social relations, resulting in distress, suffering and manifestation of disease (Farmer, 1996, p. 272).

The federal and Ontario provincial legislation, policies, and institutional practices enacted to regulate healthcare delivery restrict access to public healthcare coverage and essential health services for people identified as medically uninsured (Goldring et al., 2009; Hartog, 2020; MOHLTC, 2020c). The expectation is that medically uninsured individuals would cover healthcare costs out-of-pocket, have employment-based insurance plans, or purchase private insurance. However, medically uninsured Black immigrants in this study were precariously employed or unemployed and on social welfare, and therefore could not purchase private insurance or afford to pay for HIV healthcare and treatment out-of-pocket. Under the Ontario health legislation, immigrants who receive permanent residency or individuals who move to Ontario from another province are subjected to a 3-month waiting period before accessing healthcare through the public health insurance plan. The 3-month waiting period delayed or interrupted HIV healthcare for Black immigrants (Angus et al., 2013). British Columbia, Manitoba, and Quebec are the only Canadian provinces that still apply the 3-month waiting period (Goel et al., 2013). While the Ontario government temporarily removed the 3-month waiting period for OHIP coverage to be reinstated at a future date to mitigate the health-related consequences of COVID-19, the temporality of the policy change has resulted in fear and uncertainty for precarious status immigrants (Edmonds & Flahault, 2021).

Findings also revealed how Canada’s legislation regulating immigration and healthcare contributed to gatekeeping of healthcare and policing of medically uninsured immigrants, in an already exclusionary and violent system. Healthcare providers and administrative staff involved in gatekeeping work enforced policies and bureaucratic procedures to determine eligibility and allow or deny access to healthcare settings and services (Pollock et al., 2019). Gatekeeping compromised healthcare providers’ duty of care and standards of practice by relegating them to institutional gatekeepers. Furthermore, gatekeeping practices coupled with stigma, discrimination and systemic and anti-Black racism amounted to long wait times, denial of care and inadequate and suboptimal care within healthcare settings (Drewniak et al., 2017). Although medically uninsured individuals are entitled to emergency medical care in Canada, complex policies, administrative procedures and discriminatory practices that shape gatekeeping work presented barriers to accessing HIV healthcare. Emergency department visits and hospitalization resulted in expensive out-of-pocket medical bills and higher healthcare costs. The findings complement scholarships that points to the impact of inequities in structural determinants and intersecting forms of structural violence, which have been linked to limited health care access and poor health outcomes for immigrants (Houston et al., 2021).

Black immigrants and healthcare workers foregrounded the workaround processes of seeking healthcare through CHCs and independent doctors, and the immigration work of applying for legal permanent status in Canada as essential components of HIV healthwork. These workaround processes involved interactions with institutional systems and networks of actors, including healthcare workers such as nurses, physicians, pharmacists, social workers in shelters, CBOs and ASOs and immigration lawyers. The CHCs are non-profit organizations that receive funding from the MOHLTC to provide pre-defined primary healthcare to marginalized community members who are medically uninsured. While the Ontario government introduced the CHC model of care to address legislative gaps within the broader healthcare system and barriers experienced by medically uninsured people, there are still structural inequities that restrict access and provision of adequate healthcare for medically uninsured individuals. The CHCs remain inaccessible to many Black immigrants because of long waitlists, capacity limitations, and strict eligibility criteria for accepting new patients (Caulford & D'Andrade, 2012). Furthermore, the CHCs are underfunded and lack the resources necessary for providing adequate healthcare services to precarious status immigrants (Caulford & D'Andrade, 2012; Caulford & Vali, 2006). As a result of limited resources, the CHCs do not provide prescription medications, including ART and cover healthcare-related costs such as laboratory tests and examinations. The CHCs also frequently experience a shortage of healthcare providers because of limited funding. Additionally, some healthcare providers shy away from providing care because of the complexities of the CHCs billing system. Due to these structural barriers, medically uninsured Black immigrants spend much time doing the healthwork of looking for healthcare workers, seeking referrals, waiting, self-advocating and undergoing the emotive work of disclosing their HIV status. They rely on ASOs and CBOs who have established social networks with the CHCs and medical practitioners to negotiate access to healthcare and initiate the intake process. Healthcare workers engaged in the complex work of making referrals and negotiating access to HIV healthcare and compassionate medication for Black immigrants. The work of negotiating care is emotional, tedious and time consuming, leading to avoidance and delays in accessing healthcare. 

Therefore, immigration and healthcare legislation of right to health, lack of knowledge of how Canada’s healthcare system is organized and how to go about accessing healthcare services, absence of health insurance coverage, unemployment, high cost of HIV treatment, gatekeeping practices, CHCs' complex administrative processes, systemic and anti-Black racism, and stigma and discrimination force precarious status immigrants to avoid and delay accessing HIV healthcare and medication (Miklavcic, 2011; Quinn et al., 2018). Black immigrants avoid seeking HIV healthcare for fear of surveillance, detention and deportation by immigration authorities, leading to mental health and physical harm. These structrural barriers and inequities subject Black immigrants to the emotional and violent work of waiting for emergency care and living with untreated comorbidities until their conditions deteriorate significantly or are on the verge of death. The findings demonstrated that the CHC model of care and compassionate care programs designated to deliver healthcare to medically uninsured individuals and the limited option to access public healthcare through emergency hospital visits reinforce and obscure inequities produced by unjust and exclusionary laws regulating healthcare delivery in Canada.

Immigration work of regularizing precarious status emerged as an essential healthwork activity for Black immigrants living with HIV. As Black immigrants sought access to compassionate HIV healthcare and treatment, they also commenced the process of making refugee claims and applying for permanent legal status and residency in Canada. The work of applying for permanent legal status was viewed as the legal and guaranteed pathway to accessing public healthcare in Canada and gaining access to consistent HIV healthcare and treatment. However, bureaucratic, complex and lengthy procedural immigration processing resulted in extensive delays in determining refugee claimants' eligibility status, and long waiting periods before getting parment legal status and residency. Findings also revealed that the COVID-19 pandemic disrupted the work processes of applying for permanent legal status by curtailing immigration processing and adjudication. Black immigrants with precarious status unable to make refugee claims, and those awaiting eligibility determination for their refugee claims, permanent legal status and residency remained ineligible for health coverage and other health-related resources, including HIV healthcare and treatment, work permits, drug benefits, and social assistance.

Exclusionary othering and laws that restrict access to HIV healthcare and treatment for ACB immigrants with precarious immigration status highlight the manifestation of structural violence that materialize through inequities and social injustice, causing harm, suffering and poor health outcomes. Access to healthcare and medication is not a luxury for precarious status immigrants but a fundamental human right that must be granted to all human beings and an essential determinant of individual and public health outcomes (Da Lomba, 2017; Garrod et al., 2020). Restricting access to HIV healthcare, medication and related health services undermine Black immigrants’ fundamental human rights including right to health (Sanders & Bisaillon, 2019). The absence of constitutional right to healthcare for immigrants -jeopardizes and derail public health and global efforts of reducing risks of viral transmission and ending the HIV/AIDS epidemic leaving no one behind (Assefa et al., 2020; McClarty et al., 2021). Restricting access to HIV healthcare and medication for precarious status immigrants hinders timely diagnosis, treatment and management of infectious diseases and comorbidities and access to preventative health interventions. Timely and consistent access to health services, including diagnosis, treatment, and social support is essential for improving and maintaining individual health and protecting public health (Assefa et al., 2020).

Study findings showed that alternative programs set up to work around the healthcare system gaps are insufficient to meet the needs of precarious status immigrants living with HIV in Canada. Thus, relying on alternative programs to bridge gaps created by exclusionary and bureaucratic legislative frameworks institutionalize and normalize structural violence. However, structural violence experienced by Black immigrants cannot solely be attributed to dominant Canadian laws that restrict the right to health. Whereas the right to health is not guaranteed in the Charter of Rights and Freedoms, it is also critical to contextualize the legal-medical violent conditions experienced by Black immigrants within historical and legacies of racialization, colonialism, xenophobia, and oppression and anti-Black racism, both within Canada and globally (Dryden & Nnorom, 2021).

Cognizant of the significance of the scientific advances, World Health Organization (WHO) Member States committed to universal health coverage for all irrespective of background in 2005 to ensure universal access to HIV healthcare and treatment. In 2005 during the General Assembly High-Level Meeting on HIV/AIDS, United Nations (UN) Member States committed to the goal of “universal access to comprehensive prevention programmes, treatment, care and support” and passed a resolution towards universal access to HIV/AIDS services in 2006 (United Nations, 2006). The Sustainable Development Goals (SDGs), adopted by the United Nation’s Member states in 2015, aim to keep people healthy and safe while “leaving no one behind.” One of the SDGs’ primary goals is ending the HIV/AIDS epidemic to improve the world’s population’s health and wellbeing (UN, 2015). Central to SDGs’ goal of ending HIV/AIDS is member states’ commitment to achieving universal healthcare (UHC). The SDGs define UHC as “access to quality essential healthcare services and access to safe, effective, quality and affordable essential medicines and vaccines for all” (Gostin, 2021, p. 1). However, the exclusionary othering of precarious status immigrants and their exclusion from accessing health insurance coverage and HIV healthcare undermines the SDGs of keeping people healthy and global efforts towards ending HIV while leaving no one behind.

The evidence that Black immigrants with precarious immigration status are being left behind in HIV response due to lack of health coverage and universal and comprehensive access to healthcare calls for policy and program interventions within healthcare systems that are based on basic human rights, equity and social justice approach (Etowa et al., 2020; McClarty et al., 2021). Our findings expand on a growing body of knowledge on legal and social determinants of health, demonstrating that immigration and health policies and extended state-level regulations intersect to affect immigrants’ access to HIV and treatment (Caulford & Vali, 2006; Gostin, 2021; Martinez et al., 2015). The UNAIDS report entitled *Miles to Go-Closing Gaps, Breaking Barriers, Righting Injustices *emphasize that laws and policies that promote and protect human rights are essential for improving people’s wellbeing and health, reducing people’s vulnerability to the HIV epidemic and enhancing accessibility to essential, quality and effective healthcare services (UNAIDS, 2018). Achieving the 90-90-90 targets, Fast Track goals of getting to zero transmission and SDGs goal of keeping people safe and healthy leaving no one behind require that we make policy changes that address structural violence embedded within social, legal and medical structures (UNAIDS, 2014a, 2014b). This involves revamping immigration and healthcare policies to ensure the right to health, including universal access to healthcare and treatment.

Therefore, governments must embark on specific legislative reforms and policy actions that obligate governments to provide adequate and equitable healthcare for all without discrimination. Immigrants with precarious immigration status do not have a total lack of rights but have fundamental human rights, including the right to healthcare acknowledged by international human rights law (Da Lomba, 2017). The ICESCR introduced the “minimum core obligation” concept to ensure “the satisfaction of, at the very least, minimum essential levels of each of the rights is incumbent upon every State party” (ICESCR, 2000). Paragraph 43 of the General Comment 14 on the right to health indicates that states have the obligation “to ensure the right of access to health facilities, goods and services on a non-discriminatory basis, especially for the vulnerable or marginalized group (ICESCR, 2000). Additionally, States have a minimum obligation to provide essential medicines. The WHO Action Programme on Essential Drugs defines essential medicines as “those that satisfy the priority health care needs of the population” based on disease prevalence and evidence of their efficacy and safety. Furthermore, extending the right to health to include public health, states that are party to the ICESCR are expected to prevent, treat, and control epidemics, endemics, occupational and other diseases, including creating conditions that enable access to medical services and care for everyone. States are also required to adopt and implement public health strategies and plans to address the health concerns of the general population. Despite the commitment to minimum core obligation, policy support for ensuring universal healthcare access remains an illusion for precarious status immigrants.

Therefore, Canada must meet the commitment in the ICESCR and SDGs goal on UHC to successfully achieve the national and global targets of ending the HIV/AIDS pandemic without leaving precarious status immigrants behind and protecting public health. A healthcare system that provides UHC and ensures access to equitable and high-quality health services for all is crucial for securing individual and public health, particularly in the era of syndemics such as HIV/AIDS and COVID-19 (Legido-Quigley et al., 2019). Extending the right to health and ensuring access to healthcare to precarious status immigrants through UHC requires a commitment and activation of the principles of universality, accessibility, equity, affordability, cost-effectiveness, and high-quality services articulated in CHA. The CHA takes an equity lens stating in the preamble that “continued access to quality healthcare without financial or other barriers will be critical to maintaining and improving the health and wellbeing of Canadians” ("Canada Health Act, 1985"). Furthermore, CHA’s principle of universality and accessibility is embedded in the federal health plan to promote Canadian intrinsic values of equity and solidarity. Legislative reforms should also involve removing all healthcare barriers, including the 3-month waiting period, and enacting universal healthcare access and a comprehensive national pharmacare program accessible by all irrespective of immigration status and socio-economic status (Hajizadeh & Edmonds, 2020). Thailand is the only country that provides unconditional access to UHC to everyone, including undocumented immigrants (Onarheim et al., 2018).

## Conclusion

This IE explored how legislation and institutional practices governing healthcare delivery in Canada organize and constrain HIV healthcare and treatment work of Black immigrants with precarious status and living with HIV in Canada. Timely and consistent access to HIV healthcare and health-related services is essential for all people living with HIV in Canada, whether Black immigrants with precarious status or Canadian citizens. However, healthcare in Canada is organized, regulated, and delivered in discriminatory ways, which violates the rights of precarious status immigrants living with HIV. Immigration and residency statuses are critical determinants of eligibility and access to healthcare under federal and provincial health insurance schemes. Legislation and institutional practices regulating healthcare delivery in Canada exclude precarious status immigrants from public healthcare insurance plans and restrict their ability to access healthcare services within a publicly funded healthcare system. These exclusionary and discriminatory legislative frameworks present barriers to timely linkage and engagement in HIV healthcare and treatment for precarious status immigrants. The inquiry illuminates existing gaps and opportunities to enhance HIV standardized best practices of timely linkage and engagement in HIV healthcare and treatment. Successfully achieving the national and global goals of ending HIV/AIDS, leaving no one behind, relies on governments’ willingness and commitment to include precarious status immigrants living with HIV in the design and implementation of healthcare insurance plans and HIV prevention policies. Governments must strive to ensure that immigration and healthcare systems are not discriminatory and do not violate the right to health.
